# Limb Salvage Resection of Massive Dedifferentiated Thigh Liposarcoma in a Patient Lost to Follow-Up

**DOI:** 10.7759/cureus.13356

**Published:** 2021-02-15

**Authors:** Nicholas J Calvo, Adam J Mann, Miguel Lopez-Viego, Thomas Genuit

**Affiliations:** 1 Surgery, Florida Atlantic University Charles E. Schmidt College of Medicine, Boca Raton, USA; 2 Surgery, Bethesda Hospital East, Baptist Health South Florida, Boynton Beach, USA

**Keywords:** dedifferentiated liposarcoma, extremity liposarcoma, dedifferentiated extremity liposarcoma, limb-salvage surgery, soft tissue sarcoma, well-differentiated liposarcoma

## Abstract

Dedifferentiated liposarcoma (DDL) is a rare soft tissue tumor that represents a non-lipogenic progression of well-differentiated liposarcoma (WDL). Unlike WDL, DDL has the propensity for metastasis and is associated with an increased incidence of local recurrence. For DDL of the extremities that is resectable with acceptable functional outcomes, treatment includes primary surgical resection with negative margins. Although rare due to advances in reconstructive techniques, amputation for DDL of the extremities should be considered in which appropriate tumor resection cannot be performed without adequate preservation of limb function. We present the clinical progression of a patient with a large DDL of the right thigh who was initially lost to follow-up, but ultimately underwent delayed definitive therapy with the intent for limb salvage. This case illustrates the importance of assessing neurovascular, osseous, and soft tissue involvement to properly predict and preserve limb function while achieving adequate tumor resection.

## Introduction

Liposarcomas are rare soft tissue tumors that account for a worldwide incidence of 0.7 cases per 100,000 persons per year, but they are among the most common types of soft tissue sarcomas (15%) [1]. Well-differentiated liposarcoma (WDL) and dedifferentiated liposarcoma (DDL) comprise 50-70% of liposarcomas. DDL represents a non-lipogenic progression of WDL that is often found in association with precursor WDL tumors. DDL is associated with a worse prognosis and decreased survival due to its propensity for metastasis and increased incidence of local recurrence [2,3]. Management of localized liposarcomas includes radical surgical resection with clear margins, as well as adjuvant or neoadjuvant radiation. Rates of local and/or distant therapy failure and prognosis are primarily related to age at diagnosis, tumor location and size, stage at initial presentation, histologic grade, heterologous differentiation, completeness of initial resection, need for contiguous organ resection, and primary anatomical site [4-7]. Notably, poorer outcomes are associated with retroperitoneal tumors [6].

We present a patient with a large DDL of the right thigh. The patient was initially lost to follow-up, but eventually underwent delayed definitive therapy with the intent of limb salvage. This case illustrates the clinical progression of a DDL of the thigh and the importance of assessing neurovascular, osseous, and soft tissue involvement to properly predict and preserve limb function while achieving adequate tumor resection.

## Case presentation

**Figure 1:**
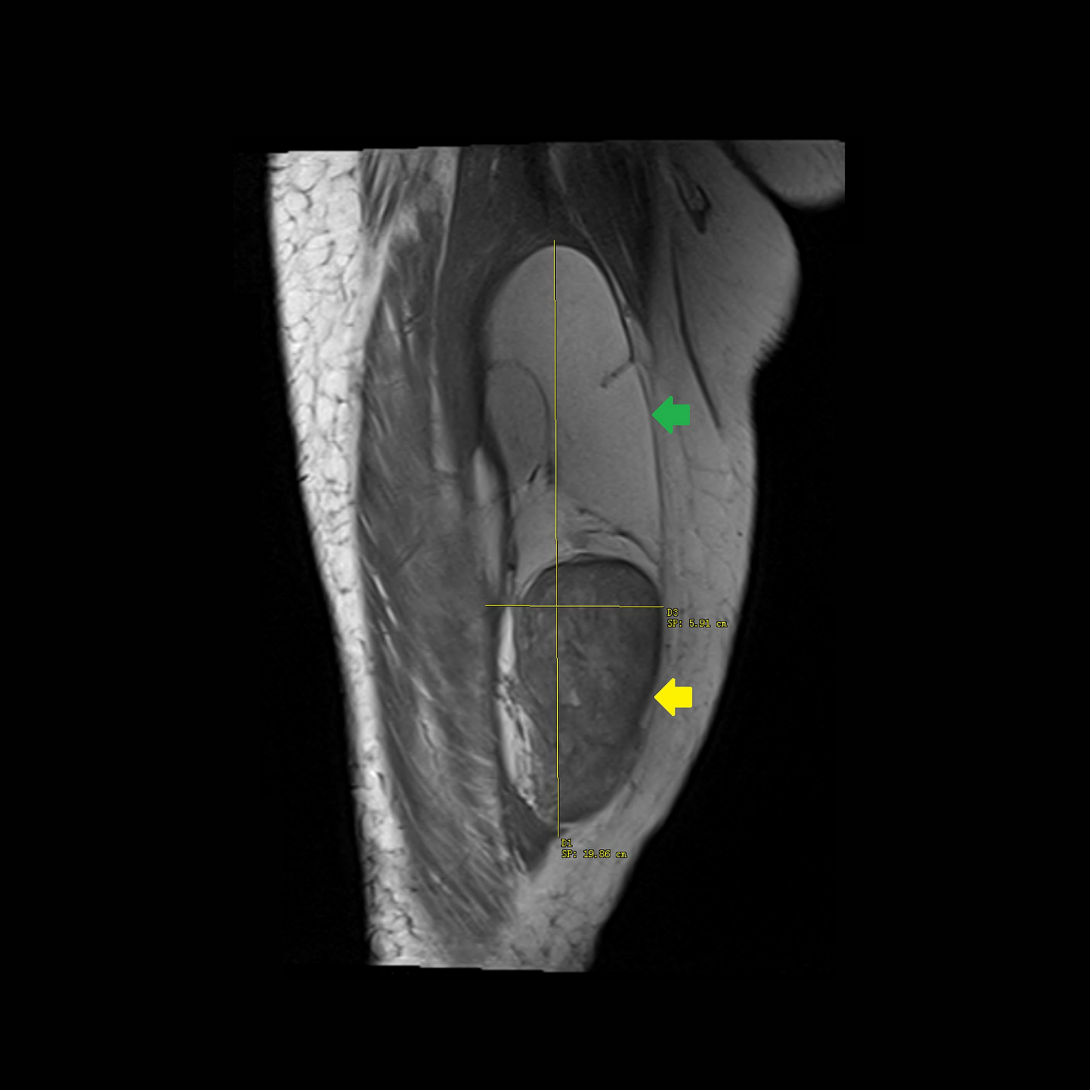
T1-weighted turbo spin echo MRI without contrast of the right thigh in coronal plane. A 20 × 6 × 5 cm multi-loculated mass with a superior lipogenic component (green arrow) and inferior non-lipogenic component measuring 9 × 5 × 5 cm (yellow arrow). MRI, magnetic resonance imaging

**Figure 2:**
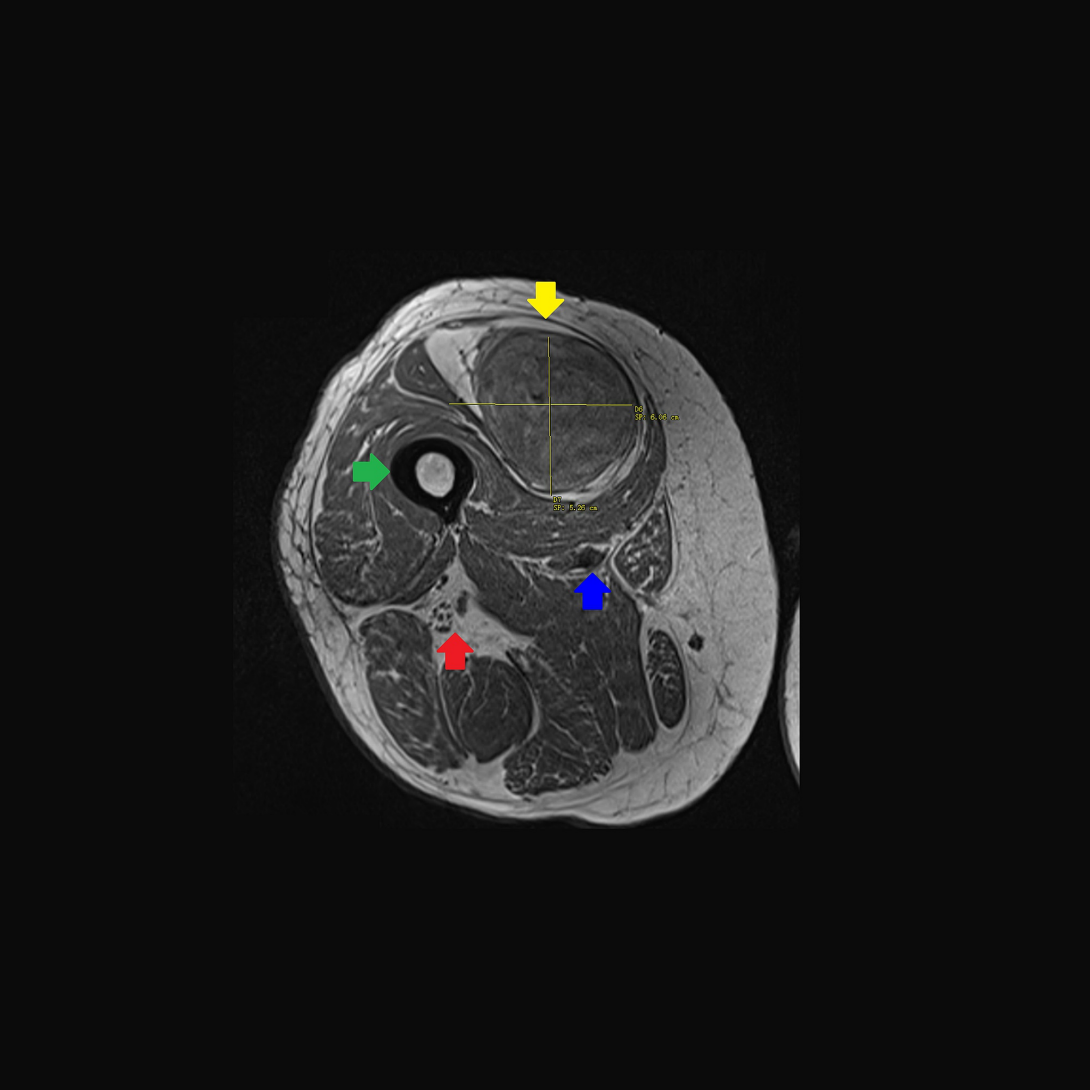
T1-weighted turbo spin echo MRI without contrast of the right thigh in axial plane. A 20 × 6 × 5 cm multi-loculated mass (yellow arrow) with a superior lipogenic component and inferior non-lipogenic component (9 × 5 × 5 cm). There is no invasion of the femur (green arrow), profunda femoris artery/vein (red arrow), or superficial femoral artery/vein and posterior division of the femoral nerve (blue arrow). The tumor displaces the rectus femoris medially and the vastus intermedius posteriorly. MRI, magnetic resonance imaging

A 70-year-old female patient with a past medical history of hypertension, hyperlipidemia, diabetes mellitus, and obesity presented to the emergency department with a painful anterior right thigh mass measuring approximately 25 cm in length. At an outside facility 2.5 years prior, magnetic resonance imaging (MRI) without contrast of the right thigh identified a heterogeneous mass with a lipogenic and a non-lipogenic component measuring 20 × 6 × 5 cm (Figures 1, 2). According to the medical record, the mass had been present for years without significant changes and was causing no symptoms. An incisional biopsy of the mass, at that time, revealed adipose tissue with fibrous septations and atypical cells with enlarged hyperchromatic nuclei. Fluorescence in situ hybridization (FISH) showed MDM2 gene locus amplification, but tests for CDK4 and p16 were not performed. The biopsy and imaging findings were suggestive of DDL, rather than other lipomatous tumors (i.e., WDL, lipoma, dysplastic lipoma, spindle cell lipoma, or inflammatory myofibroblastic tumor). However, the patient was discharged with instructions to follow-up with surgery and medical oncology but was lost to follow-up.

**Figure 3:**
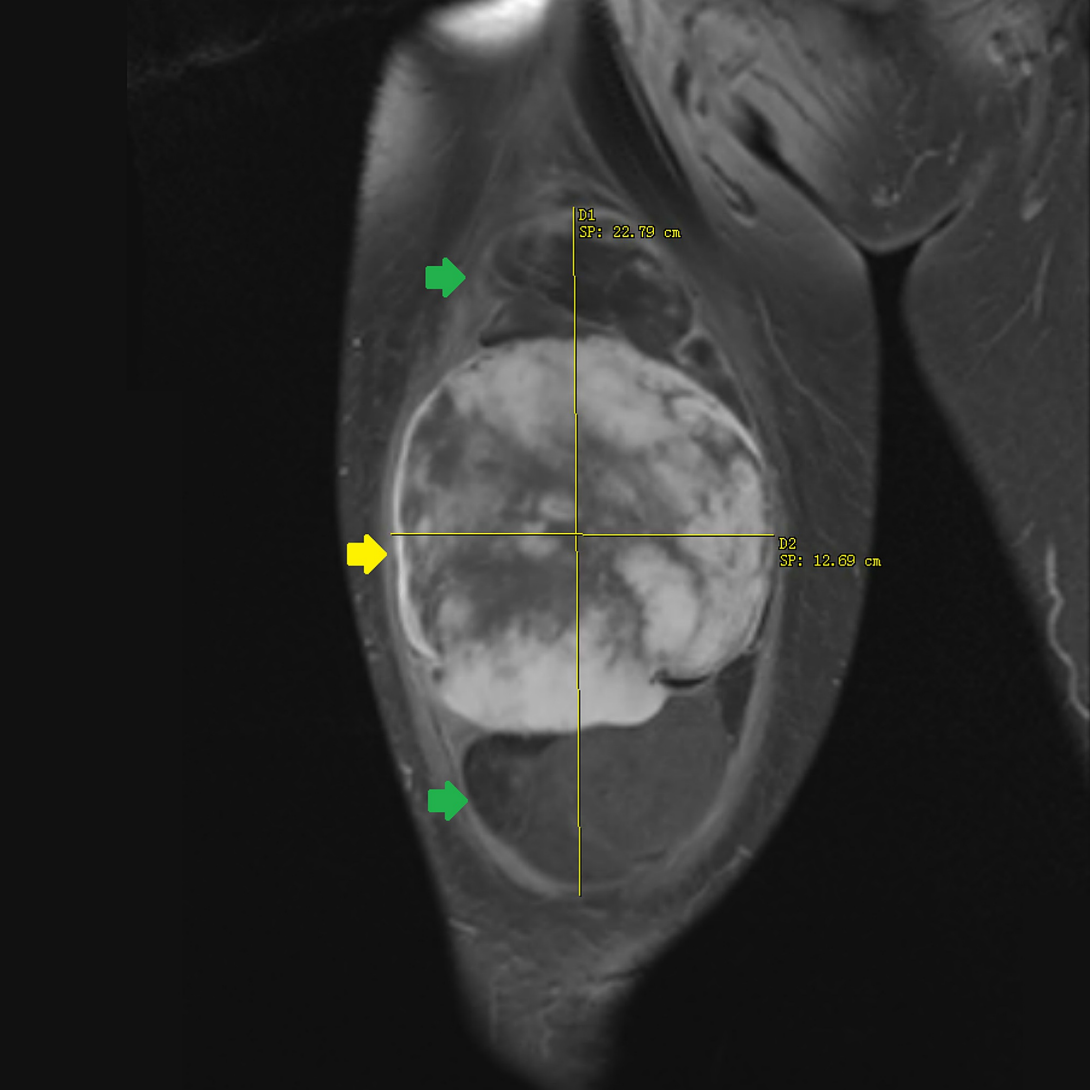
T1-weighted turbo spin echo MRI with gadolinium contrast of the right thigh in coronal plane. A 23 × 12.5 × 8.5 cm heterogeneous mass with a peripheral fat component (green arrows) and central non-lipogenic enhancing component measuring 12.6 × 12.6 × 8.3 cm (yellow arrow). MRI, magnetic resonance imaging

**Figure 4:**
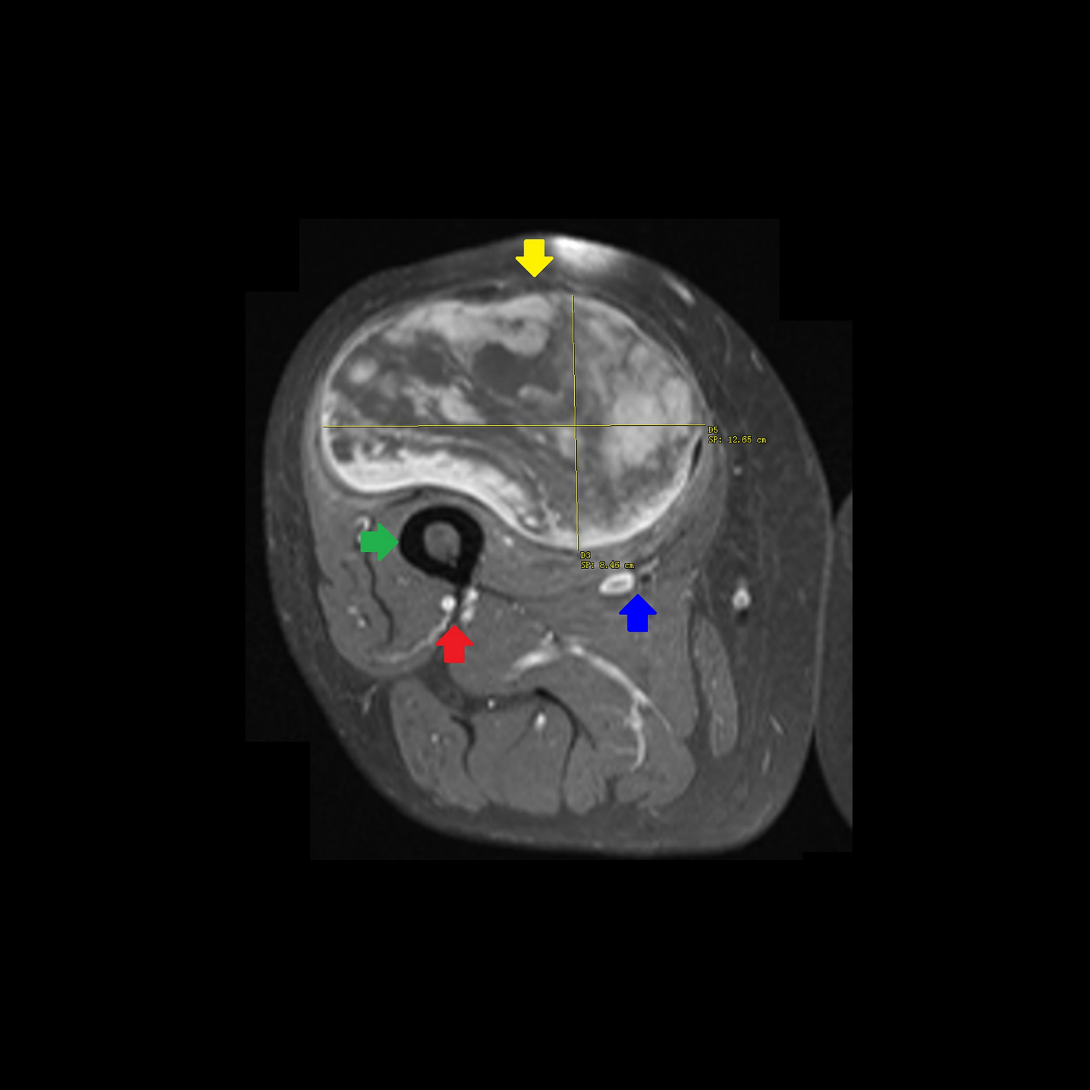
T1-weighted turbo spin echo MRI with gadolinium contrast of the right thigh in axial plane. A 23 × 12.5 × 8.5 cm heterogeneous mass (yellow arrow) abutting but not infiltrating the femur (green arrow). There is a peripheral fat component and central non-lipogenic enhancing component measuring 12.6 × 12.6 × 8.3 cm. There is no invasion of the profunda femoris artery/vein (red arrow), or superficial femoral artery/vein and posterior division of the femoral nerve (blue arrow). The rectus femoris muscle is partially encased by the tumor. The sartorius and vastus muscles are partially encased and posteriorly displaced by the tumor. MRI, magnetic resonance imaging

Subsequently, the patient presented to an outside facility six months prior to the current hospitalization with enlargement of the thigh mass, moderate pain, tingling, and decreased motor strength in the right anterior thigh compartment. An MRI with gadolinium contrast of the right thigh revealed enlargement of the mass to 23 × 12.5 × 8.5 cm with a centrally enhancing non-lipogenic component and peripheral lipogenic components (Figures 3, 4). In accordance with the guidelines for management of liposarcomas, she was referred to a tertiary care center with a multidisciplinary sarcoma team for management, but she was again lost to follow-up.

**Figure 5:**
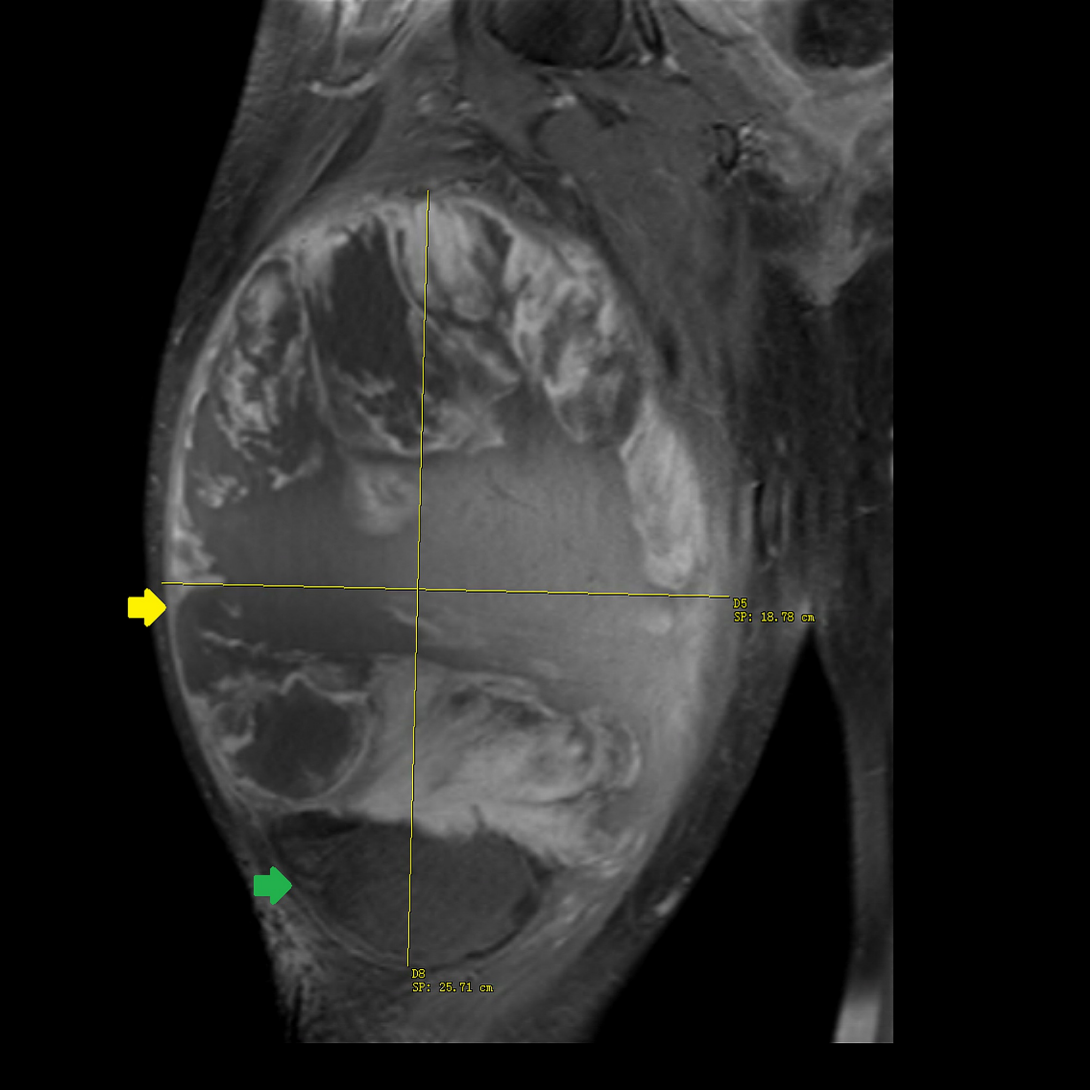
T1-weighted turbo spin echo MRI with gadolinium contrast of the right thigh in coronal plane. An anterior compartment mass measuring 26 × 19 × 14 cm. The central non-lipogenic component measures 24 × 19 × 14 cm (yellow arrow) and demonstrates irregular, inhomogeneous nodular peripheral enhancement. Peripheral fat densities are present (green arrow). MRI, magnetic resonance imaging

**Figure 6:**
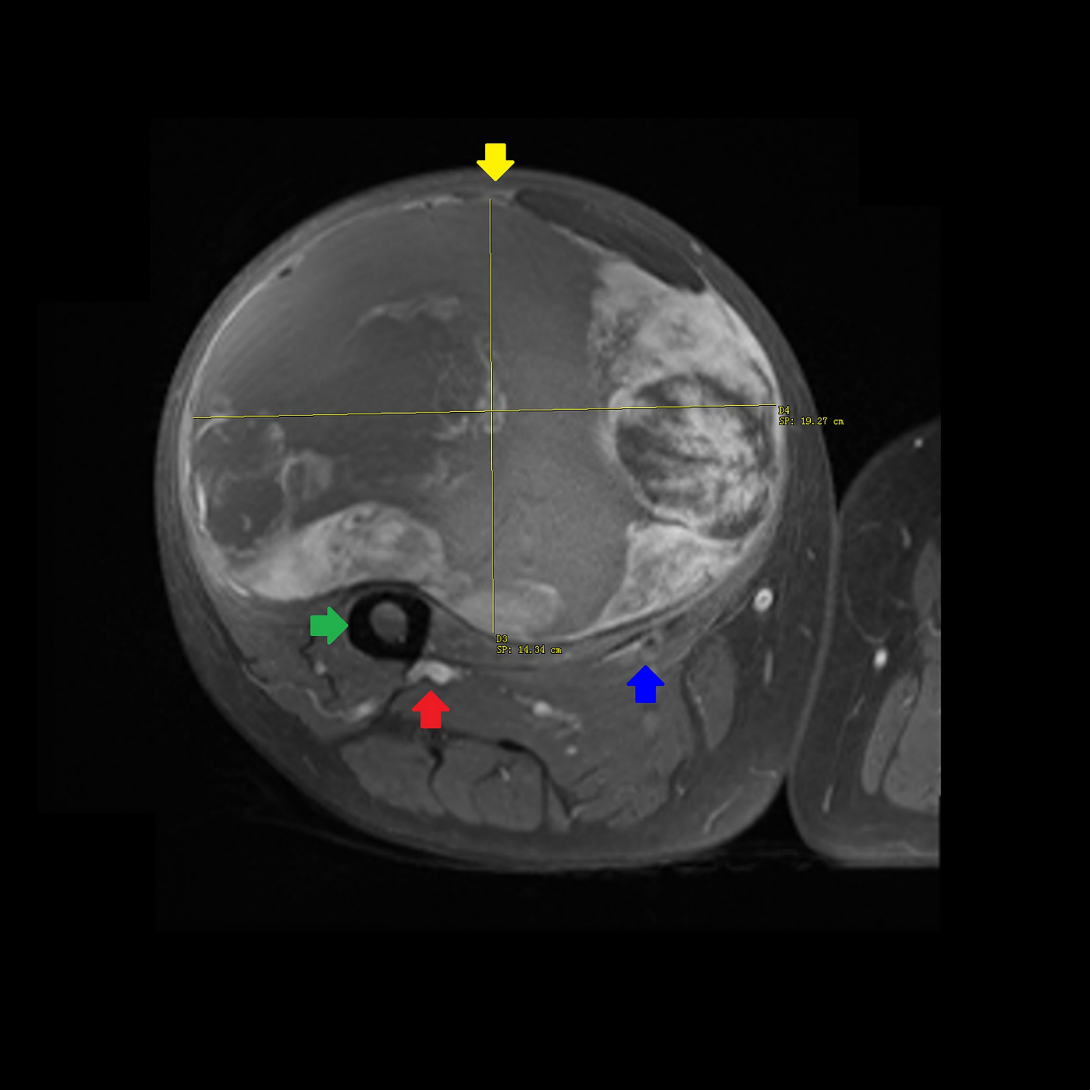
T1-weighted turbo spin echo MRI with gadolinium contrast of the right thigh in axial plane. An anterior compartment mass (yellow arrow) measuring 26 × 19 × 14 cm. The central non-lipogenic component measures 24 × 19 × 14 cm and demonstrates irregular, inhomogeneous nodular peripheral enhancement. Peripheral fat densities are present. There is no invasion of the femur (green arrow), profunda femoris artery/vein (red arrow), or superficial femoral artery/vein and posterior division of the femoral nerve (blue arrow). The rectus femoris muscle is completely encased by the tumor. The sartorius, vastus medialis, and vastus intermedius muscles are almost completely encased by the tumor. The vastus lateralis is partially encased and posteriorly displaced by the tumor. There is no tumor involvement beyond the anterior thigh compartment.

During the current hospitalization, the patient presented to the emergency room with deteriorating right thigh function, severe pain, paresthesia, and diminished muscle strength. The patient denied fever, weight loss, and/or any other significant symptoms. The patient was in distress due to the pain in her right thigh. The anterior right thigh mass measured 25 cm in length and was firm and tender to palpation. Range of motion was limited due to pain in the right lower extremity at the hip to approximately 30-105 degrees and at the knee to approximately 75-105 degrees. Muscle strength was also diminished in the right lower extremity with respect to hip flexion, hip extension, knee flexion, and knee extension. The patient had full range of motion and muscle strength at the left lower extremity and the remaining joints of the right lower extremity, as well as intact sensation and pulses in both lower and upper extremities. Inguinal lymph nodes were soft, mobile, and <1 cm in diameter. The remainder of the examination was unremarkable. MRI with gadolinium contrast showed enlargement of the right thigh mass to 26 × 19 × 14 cm (previously measuring 23 × 12.5 × 8.5 cm six months ago) with inhomogeneous nodular enhancement and peripheral fat densities (Figures 5, 6). There was no involvement of the femur, profunda femoris artery/vein, posterior division of the femoral nerve, and/or superficial femoral artery/vein (Figure 6). The rectus femoris muscle was completely encased by the tumor. The sartorius, vastus medialis, and vastus intermedius muscles were nearly completely encased by the tumor. The vastus lateralis was partially encased and posteriorly displaced by the tumor. There was no tumor involvement beyond the anterior thigh compartment. There was no inguinal or pelvic lymphadenopathy. Computed tomography (CT) of chest, abdomen, and pelvis with contrast showed no metastases or other acute processes.

Treatment

Despite the tumor’s significant size, it was determined that limb salvage should still be possible as there was no neurovascular encasement or metastatic disease and tumor resection could likely be achieved with adequate preservation of right lower extremity function. Given the patient’s history of medical non-compliance with follow-up, the decision was made to proceed with surgical intervention during this admission rather than referral to another facility with a multidisciplinary sarcoma team [8].

**Figure 7:**
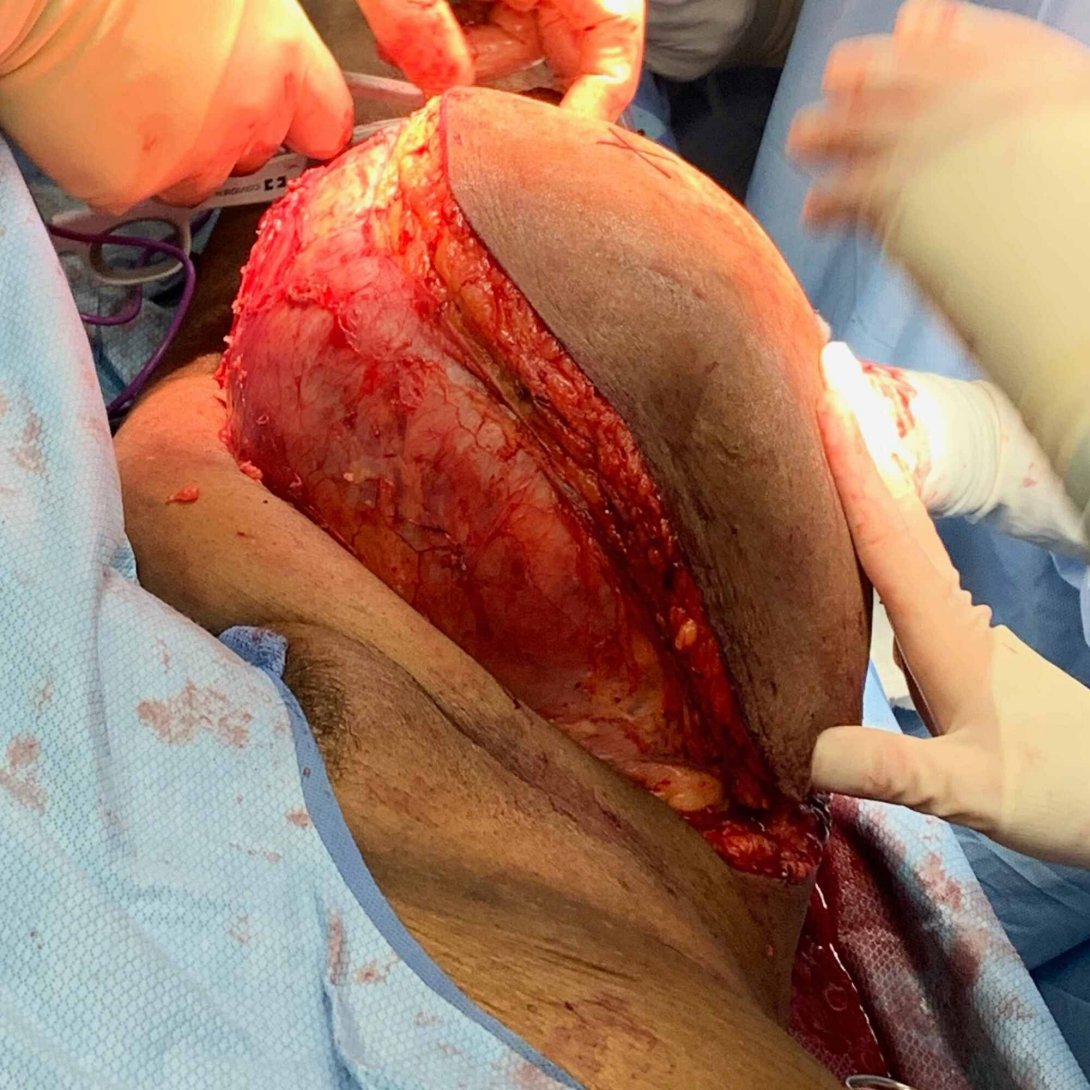
Tumor resection of the anterior right thigh mass.

**Figure 8:**
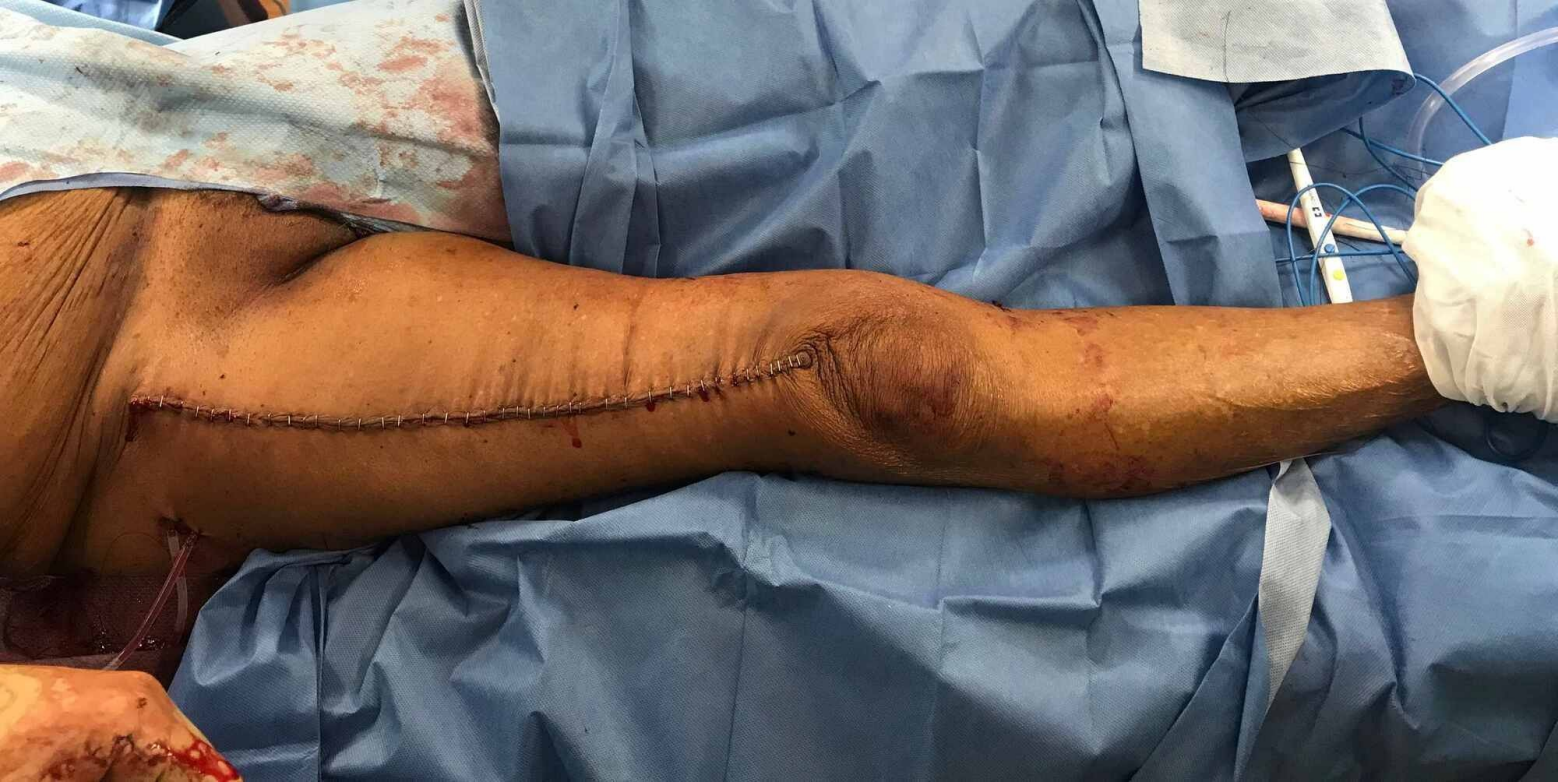
Primary closure of the anterior right thigh.

After sedation and intubation, the patient was placed in a supine position and her right lower extremity was draped and prepped above the ankle. An elliptical incision was made around the palpable border of the mass and the well-circumscribed, lobulated, soft, rubbery mass was dissected with a margin of surrounding soft tissue/muscle. Intraoperative evaluation revealed that the tumor completely invaded the rectus femoris and almost completely invaded the sartorius, vastus medialis, and vastus intermedius muscles. This portion of the tumor was resected en bloc with a margin of the surrounding muscle which did allow for the preservation of some musculature. An unidentified branch of the femoral nerve (most likely the branch to the rectus femoris) was completely encased within the tumor and was resected en bloc (Figures 7, 8). Medially the tumor was adjacent to the superficial femoral artery but without encasement or direct invasion, allowing its preservation. The tumor did not extend past the anterior thigh compartment. Intraoperative frozen section of the deep margins showed no malignancy.

**Figure 9:**
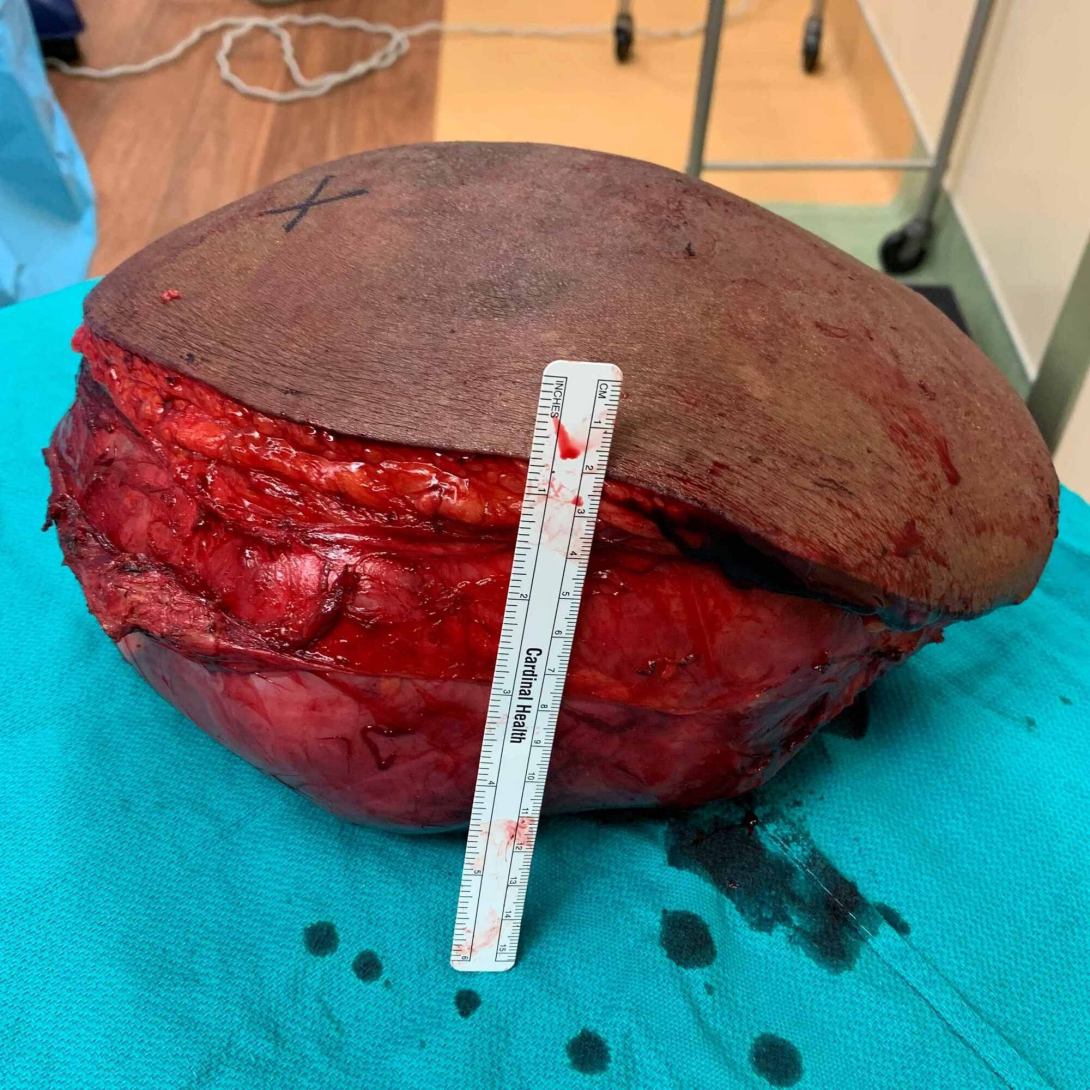
Macroscopic tumor appearance: anterior-medial view.

**Figure 10:**
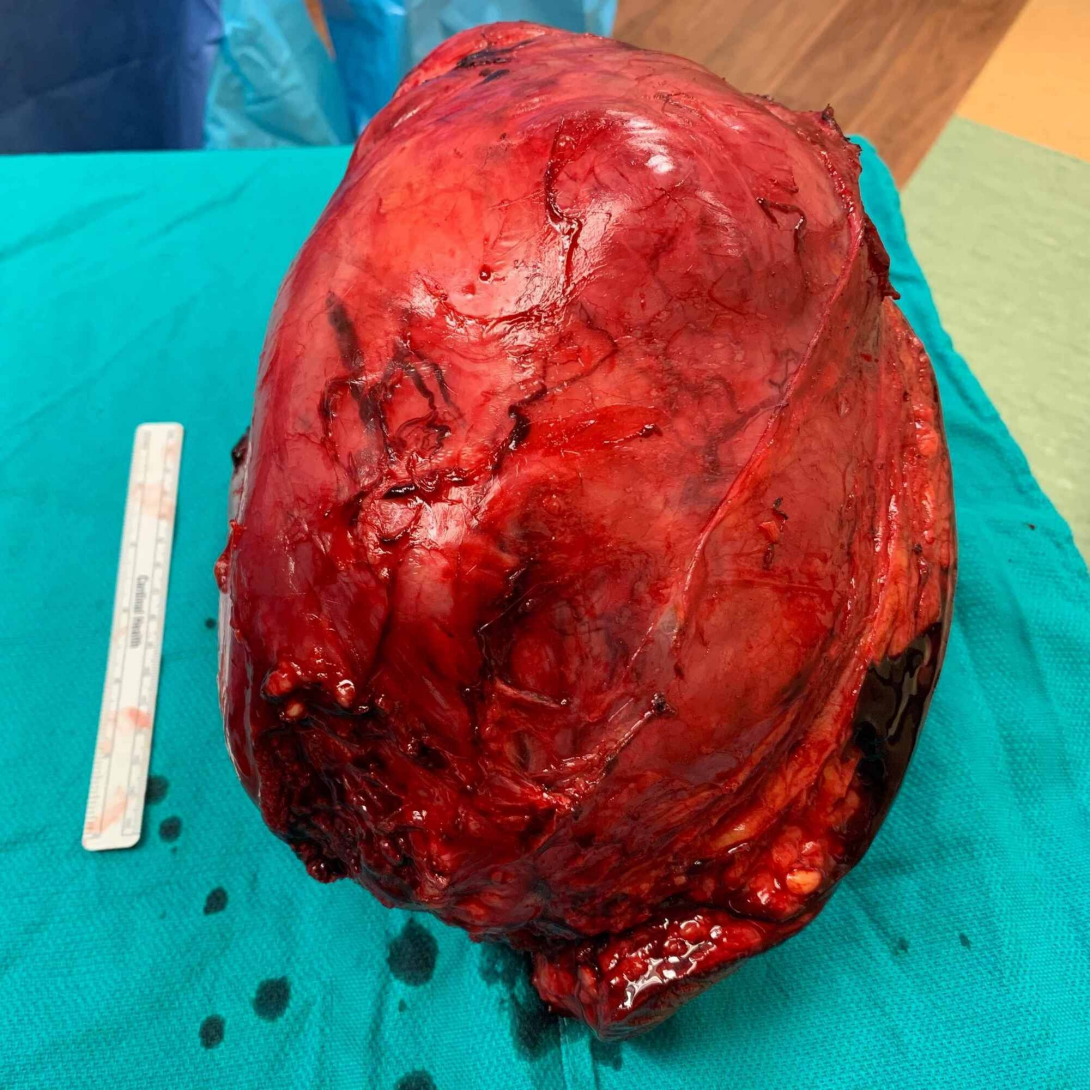
Macroscopic tumor appearance: medial view.

**Figure 11:**
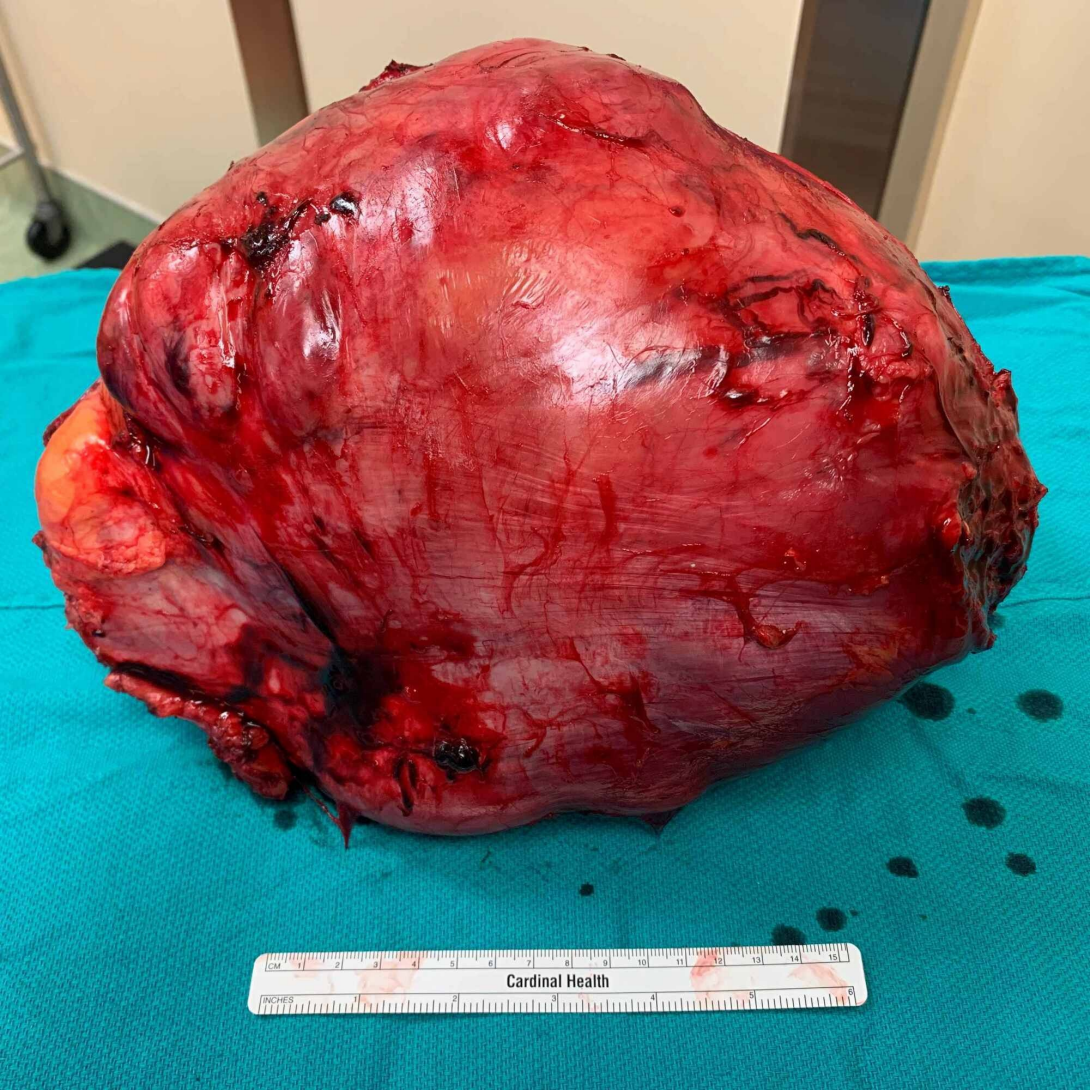
Macroscopic tumor appearance: posterior view.

**Figure 12:**
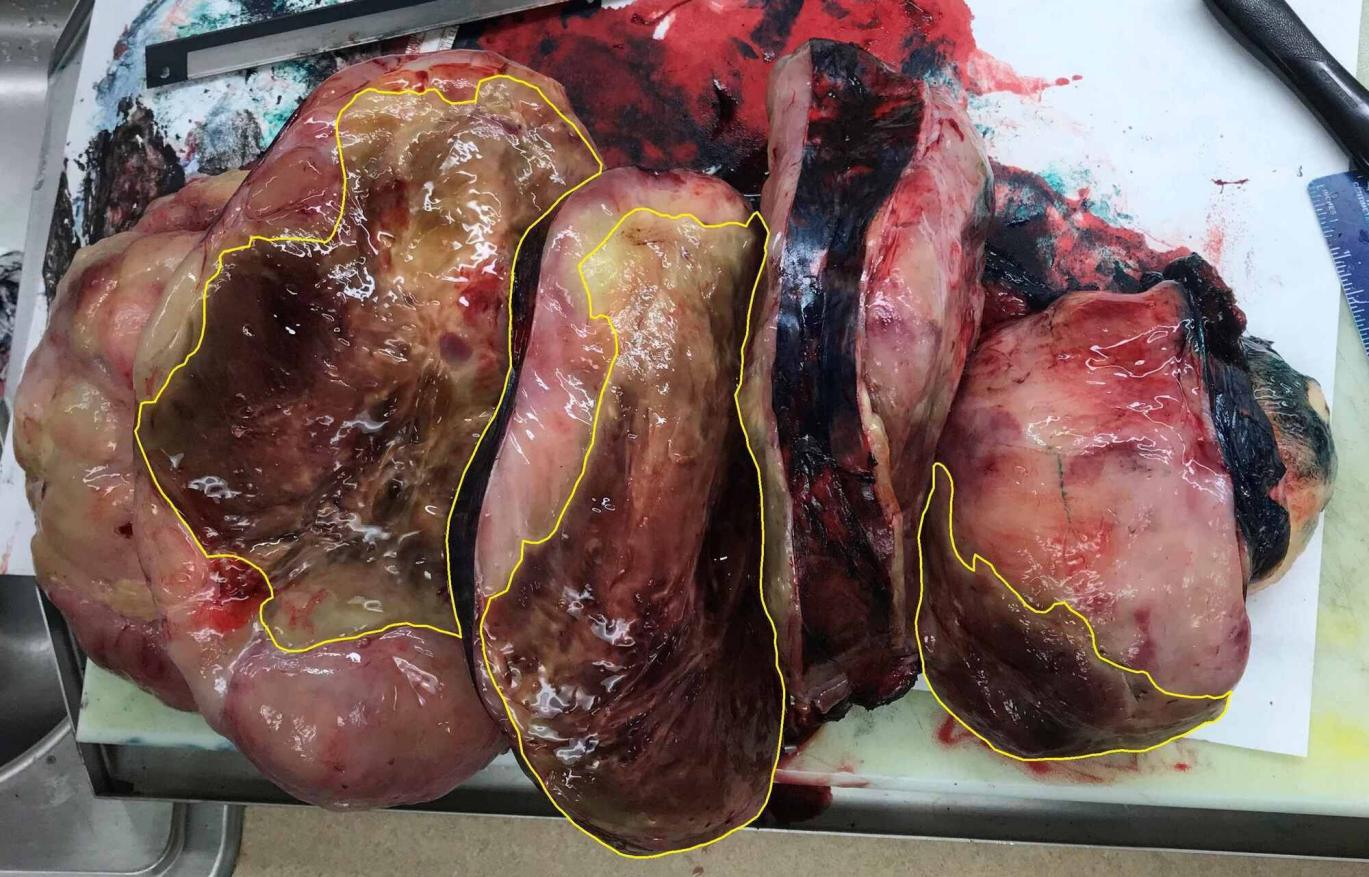
Longitudinal cross-sections of the tumor. Sectioning revealed a marbled fatty surface with areas of necrosis. There is a central lobulated mass measuring 20 × 15 × 14 cm. Within the central mass are firm and fibrotic nodular areas intermixed with foci of cystic degeneration and necrosis (yellow outlines).

Pathologic examination revealed the mass to measure 30 × 20 × 15 cm and weigh 4,607 g (Figures 9-11). Sectioning revealed a marbled yellow cut surface with interspersed cystic and firm/fibrotic white areas (Figure 12). Fat necrosis was seen along the periphery. No lymph nodes were identified. The central non-lipogenic component of the tumor measured 20 × 15 × 14 cm with a mitotic rate of five mitoses per 10 HPF and was classified as Fédération Nationale des Centres de Lutte Contre le Cancer histologic grade 2. The tumor was classified as American Joint Committee on Cancer pathologic stage III (T4N0M0G2). Scattered necrosis was present in 10% of the tumor. All margins were negative for tumor invasion, with the closest margin being <1 mm, posteriorly. MDM2 gene amplification was positive by FISH. These findings indicated a DDL of the right anterior thigh.

Outcome

The patient’s post-operative recovery was uneventful. On post-operative day 2, her range of motion in the right hip improved to 15-120 degrees (from 30-105 degrees) as well as in the right knee to 30-120 degrees (from 75-105 degrees). Muscle strength in the right lower extremity improved with respect to hip flexion, hip extension, knee flexion, and knee extension. By post-operative day 4, the patient demonstrated continued improvement in right knee extension and was able to ambulate with a rolling walker. The patient was discharged on post-operative day 5 and closely followed throughout completion of her adjuvant radiotherapy and physical therapy. The patient received intensity-modulated radiation therapy to the anterior right thigh with bolus to the surgical scar. Over eight weeks, 61.2 Gray in 34 fractions was administered to both the superior and inferior portions of the anterior right thigh. Based on the surgical margin status, the patient received an additional 11.2 Gray of radiation to the 50 Gray recommended for patients without pre-operative radiation therapy [8]. This was well-tolerated except for hyperpigmentation and mild desquamation of her skin. Despite the narrow margins, the primary resection combined with radiotherapy was deemed sufficient treatment for the patient’s liposarcoma. At one month post-operatively, the patient was able to ambulate with the assistance of a cane.

## Discussion

Liposarcomas are soft tissue tumors that are generally classified into four subtypes: WDL, DDL, myxoid liposarcoma, and pleomorphic liposarcoma [4]. Both WDL and DDL commonly arise in the extremities and retroperitoneum but rarely in the mediastinum, para-testicular region, or spinal cord [2,3]. Liposarcomas located in the retroperitoneum are often diagnosed at a later stage compared to those in extremities and other superficial soft tissues, which may explain to some extent the higher rates of local recurrence and metastasis of retroperitoneal tumors [2,3].

DDL is a non-lipogenic sarcoma that is thought to arise as a progression of WDL [2]. The risk of dedifferentiation to DDL is directly related to the location and duration of tumor growth [2]. About 10% of DDL arise as a recurrence of WDL, while nearly 90% of DDL occur within a primary WDL lesion, most commonly in the retroperitoneum [2,5]. Unlike WDL, DDL has a propensity for metastasis, and a greater incidence of local recurrence after resection, resulting in decreased overall and disease-free survival (Table 1) [9]. Mortality from DDL is most often due to uncontrolled local recurrent disease than metastasis [2]. However, metastasis is an indicator of poor prognosis with a five-year survival rate of <18% in patients with stage IV tumors [6]. The most common sites of metastasis in descending order include the lungs, subcutaneous soft tissues, lymph nodes, and liver [10]. The median time to metastasis is approximately eight months in patients initially presenting with localized DDL [6]. The five-year survival rate for stage I and stage II DDL is approximately 65%, which reduces to 49% for stage III and <18% for stage IV (Table 2) [6].

**Table 1:** Local recurrence/metastatic rate and five-year overall survival for extremity liposarcoma by subtype. WDL, well-differentiated liposarcoma; DDL, dedifferentiated liposarcoma. Data adapted from Vos et al. [9]

Liposarcoma subtype	WDL	DDL
Local recurrence rate (%)	23	38
Metastatic rate (%)	3	14
5-year overall survival (%)	92	54

**Table 2:** Survival rates of DDL by AJCC stage, FNCLCC grade, and primary site. DDL, dedifferentiated liposarcoma; AJCC, American Joint Committee on Cancer; FNCLCC, Fédération Nationale des Centres de Lutte Contre le Cancer. Adapted from Gootee et al. [6]

Survival rates of DDL	5 years (%)
AJCC stage	
Stage I	63
Stage II	67
Stage III	49
Stage IV	18
FNCLCC grade	
Grade 1	69
Grade 2	65
Grade 3	45
Primary site	
Head/Neck	86
Extremities	67
Pelvis	66
Thorax/Trunk	59
Retroperitoneum/Abdomen	43

Primary surgical resection with negative margins with or without subsequent neoadjuvant and adjuvant radiotherapy is recommended for soft tissue sarcomas of the extremities, superficial trunk, and head and neck areas that are resectable with acceptable functional outcomes [8]. Resection should achieve negative margins >1.0 cm and/or intact fascial planes [8,11]. Marginal resection is associated with increased rates of local recurrence, metastasis, and decreased five-year survival (Table 3) [12]. Radiotherapy is not recommended for treatment of WDL as it is considered radio-insensitive [11,13]. DDL of the extremities >5 cm and those with close or positive margins are generally treated with adjuvant radiotherapy, especially if treatment of local recurrence would likely compromise limb function [11,13]. All patients who did not receive pre-operative radiation therapy should receive 50 Gray of radiotherapy with a boost dose of 10-16 Gray for negative margins, 16-18 Gray for microscopically positive margins, and 20-26 Gray for gross residual disease [8]. However, re-resection is preferred for patients with positive surgical margins [8]. For DDL of the retroperitoneum, radiotherapy is limited in use as it has not been shown to improve overall survival [11,13]. WDL and DDL are generally resistant to chemotherapy, but there may be a role in treatment for other subtypes of liposarcoma [2,3]. Management of DDL must always include frequent follow-up to assess for local recurrence and lung metastasis with MRI of the primary tumor site every six months and chest imaging every 6-12 months [8,11].

**Table 3:** Local recurrence/metastatic rate and five-year overall survival for liposarcoma of any type by surgical margin. Data adapted from Muratori et al. [12]

Surgical margins	Wide	Marginal
Local recurrence rate (%)	7	25
Metastatic rate (%)	12	39
5-year overall survival (%)	92	75

Evidence-based recommendations for limb salvage versus amputation is limited in patients with extremity liposarcomas. Generally, amputations should be considered for patients in which appropriate tumor resection cannot be performed without adequate preservation of limb function (large tumor size, significant involvement of more than one muscle compartment, multifocality, neurovascular, and osseous involvement) [8,14-16]. Amputation of extremities with soft tissue sarcoma has not been shown to improve survival, likely due to similar rates of distant metastasis [14,15]. This combined with advances in extremity reconstructive techniques and the use of orthotic devices have minimized functional deficits and the need for amputation in the past 5-10 years to 5% [8,15,17].

In this case, there were some indications for amputation (large tumor size, near-complete involvement of the anterior thigh compartment, and the diminished range of motion and muscle strength on physical examination). However, limb salvage was attempted due to the lack of neurovascular involvement and the partial preservation of muscles of the anterior thigh compartment. Post-operatively, the patient was able to regain sufficient right lower limb function.

## Conclusions

This is a rare case of delayed management of a large lower extremity DDL with the intent for limb salvage in a patient who was initially lost to follow-up. Assessment of neurovascular, osseous, and soft tissue involvement are essential in predicting and preserving limb function while achieving adequate tumor resection. Close clinical and imaging follow-up and adjuvant therapy may aid in managing the incidence of local recurrence and disease-free/overall survival in these patients.
